# Bleeding in patients hospitalized with acute pulmonary embolism in Brazil

**DOI:** 10.1016/j.clinsp.2024.100573

**Published:** 2025-01-06

**Authors:** Leonardo Jordan Hansen Vizzotto, Corina dos Reis Sepeda, Carlos Henrique Miranda

**Affiliations:** Division of Emergency Medicine, Department of Internal Medicine, Faculdade de Medicina de Ribeirão Preto, Universidade de São Paulo (FMRP-USP), Ribeirão Preto, SP, Brazil

**Keywords:** Pulmonary embolism, Bleeding risk, Adverse events

## Abstract

**Objective:**

Acute Pulmonary Embolism (APE) is a disease with increasing incidence worldwide. Antithrombotics are the cornerstone of the treatment. Bleeding is an adverse event related to this therapy. The objective was to evaluate the prevalence of bleeding in a sample of Brazilian patients hospitalized with APE and the impact of this complication on mortality. Additionally, the performance of some bleeding predictive scores was evaluated in this sample.

**Methods:**

A retrospective cohort study was carried out on patients hospitalized with APE from January 2009 through August 2017. The medical records of these patients were reviewed, and the bleeding recorded during hospital stay was classified according to the “Thrombolysis in Myocardial Infarction (TIMI) bleeding risk.” Five different predictive scores for bleeding after APE (RIETE, PE-SARD, VTE-BLEED, Kuijer, and ATRIA) were applied. Overall mortality at 30 days and one year were assessed.

**Results:**

One hundred fifty-nine patients were included. The prevalence of any bleeding was 36/159 (23 %), major bleeding was 10/159 (06 %), minor bleeding was 11/159 (07 %), and bleeding requiring attention was 15/159 (10 %). Only major bleeding was associated with higher mortality at one-year follow-up with a Relative Risk (RR) of 2.00 (95 % CI 1.16–3.57; *p* = 0.044). All bleeding predictive scores evaluated showed low accuracy in identifying patients at higher risk of bleeding.

**Conclusion:**

Patients hospitalized with APE in Brazil had a high prevalence of bleeding. The major bleeding increased the one-year mortality. The bleeding predictive scores assessed showed limited accuracy in identifying patients at high risk of bleeding.

## Highlights

• Antithrombotics are the cornerstone for acute Pulmonary Embolism (APE) treatment.
• Patients hospitalized with APE in Brazil had a high prevalence of bleeding.
• The major bleeding increased the one-year mortality rate.
• The bleeding predictive scores assessed showed limited accuracy in identifying high bleeding-risk patients.

## Introduction

Acute Pulmonary Embolism (APE) is a highly prevalent disease considered among the top three causes of cardiovascular mortality.^1^^,^^2^ In the United States, there are approximately 370,000 cases per year, with mortality rates ranging from 16 % to 27 %.^3^^,^^4^ There were 42,000 hospitalizations due to APE, 1500 deaths per year, and >76 million reais invested in treatment between 2015 and 2019 in Brazil.^5^ The APE incidence is increasing globally. The aging population, increase in associated comorbidities, such as obesity and cancer, and greater access to imaging tests for diagnosis justified this fact.^1^^,^^3^

Antithrombotics (heparins or direct-acting anticoagulants) must be promptly started when the diagnosis of APE is established. Reperfusion therapy with fibrinolytics is indicated in the presence of hemodynamic instability. The objective of this approach is to interrupt or reduce the clot's progression, thereby reducing morbidity, mortality, and event recurrence.^6^, ^7^, ^8^, ^9^

Unfortunately, bleeding is a common and severe adverse event associated with this therapy. Based on international literature, this complication occurs in approximately 7 % of these patients. It leads to an estimated mortality of 20 %, with the highest occurrence of this complication occurring in the first seven days of treatment.^6^, ^7^, ^8^, ^9^

However, to our knowledge, no scientific investigation evaluated the prevalence of bleeding, as well as the impact of this complication on mortality, in a sample of Brazilian patients with APE.

The early identification of patients at risk of bleeding is helpful in clinical practice because it allows for the selection of antithrombotic drugs that are more appropriate to the patient's profile and ensures more surveillance. Several clinical scores are proposed for predicting the risk of bleeding after APE; however, none of them were validated for the Brazilian population.^6^, ^7^, ^8^, ^9^, ^10^

The objective of this study was to evaluate the prevalence of bleeding in a sample of Brazilian patients with APE, as well as the impact of this complication on the mortality of these individuals. Furthermore, to evaluate the prognostic performance of some bleeding predictive scores in this sample.^11^, ^12^, ^13^, ^14^, ^15^

## Methods

A cohort, retrospective, single-center study carried out in patients with a definitive APE diagnosis admitted to the Emergency Unit of the Hospital das Clínicas of the Ribeirão Preto School of Medicine of the University of São Paulo from January 2009 through August 2017. The study was approved by the research ethics committee of this institution under number CAAE 51,979,515.9.0000.5440 and followed the guidelines of the Declaration of Helsinki.

### Participants and data

Data were collected from the medical records of patients admitted with a primary diagnosis of APE in the electronic hospital discharge sheet record using codes I26.0 (pulmonary embolism with mention of acute cor pulmonale) and I26.9 (pulmonary embolism without mention of acute cor pulmonale) according to the International Classification of Diseases version 10 (ICD-10).

The confirmation of APE diagnosis was performed through a compatible clinical presentation associated with at least one confirmatory test, which could be Computed Tomography Pulmonary Angiography (CTPA) ventilation and perfusion scintigraphy, or necropsy.

After inclusion, demographic, clinical, laboratory data, and treatment options were collected from the patient's medical records. The “Pulmonary Embolism Severity Index (PESI)” score was calculated for all patients. Five different scores predicting the occurrence of bleeding were applied for these patients: RIETE, PE-SARD, VTE-BLEED, Kuijer, and ATRIA. The criteria included in each score are shown in Table 1.

**Table 1:** Bleeding risk predictive scores after acute pulmonary embolism.

Score/Year of publication	Items of the score	Risk categories	Original goal
RIETE,^11^ 2008	Recent major bleeding (2)	Low: 0-points	DVT and/or APE
	Creatinine > 1.2 mg/dL (1.5)	Intermediate: 1‒4-points	
	Anemia (1.5)		
	Previous malignancy (1)	High: > 4-points	
	Clinical-overt APE (1)		
	Age > 75-years (1)		
Kuijer,^12^ 1999	Age ≥ 60-years (1.6)	Low: 0-points	DVT and/or APE
	Female (1.3)	Intermediate: 1‒2-points	
	Malignancy (2.2)		
		High: ≥ 3-points	
PE-SARD,^6^ 2021	Anemia (2.5)	Low: 0-points	Only APE
	Syncope (1.5)	Intermediate: 1‒2.5-points	
	Renal dysfunction (1)		
		High: > 2.5-points	
VTE-BLEED,^13^ 2016	Active malignancy (2)	Low: < 2-points	DVT and/or APE
	Male with uncontrolled hypertension (1)	High: ≥ 2-points	
	Anemia (1.5)		
	Previous bleeding (1.5)		
	Renal dysfunction (1.5)		
	Age > 60-years (1.5)		
ATRIA,^14^ 2011	Anemia (3)	Low: 0‒3-points	Non-valvular atrial fibrillation
	Renal or liver disease (3)	Intermediate: 4-points	
	Age ≥ 75-years (2)		
	Previous bleeding (1)	High: > 4-points	
	Hypertension (1)		

DVT, Deep Vein Thrombosis; APE, Acute Pulmonary Embolism.

When bleeding was present during hospitalization, it was classified according to the “Thrombolysis in Myocardial Infarction (TIMI) bleeding risk” criteria into major, minor, and bleeding requiring attention. These criteria are shown in Table 2.

**Table 2:** Bleeding Stratification according to “Thrombolysis in Myocardial Infarction (TIMI) bleeding risk”.^15^

TIMI bleeding stratification	Definition
Major	Intracranial hemorrhage;
	Fatal bleeding;
	Significant clinical bleeding (hemoglobin decrease ≥5 g/dL or hematocrit decrease ≥ 15 %).
Minor	Observable blood loss: decrease in the hemoglobin 3‒5 g/dL or decrease in the hematocrit ≥ 10 %;
	Non-observable blood loss: hemoglobin decrease ≥ 4 g/dL or hematocrit decrease ≥ 12 %.
Attention required	Observable blood loss with hemoglobin decrease < 3 g/dL or hematocrit decrease < 9 %.

The outcomes evaluated were general mortality at 30 days and one year. When this outcome did not occur during hospitalization, follow-up was conducted via telephone contact by a properly trained employee of the institution's clinical research unit.

### Statistical analysis

Categorical variables were expressed as frequency and percentage. Continuous variables with normal distribution were expressed as mean and standard deviation, and the others as median and Interquartile Range (IQR). Category variables were compared through the Chi-Square test. Two quantitative variables with normal distribution were compared through the unpaired Student's *t*-test, and two variables with another type of distribution were compared through the Mann-Whitney test. Three or more quantitative variables with normal distribution were compared through the ANOVA test, and three or more variables with another type of distribution were compared through the Kruskal-Wallis test.

The Relative Risk (RR) and its respective 95 % Confidence Interval (95 % CI) were calculated to evaluate the association between bleeding and mortality.

The accuracy of different types of scores for predicting bleeding was evaluated through the Area Under (AUC) the Receiver Operating Characteristic Curve (ROC). A convenience sample was employed and no estimative was made to determine the sample size. A two-tailed p-value ≤ 0.05 was considered significant. Stata software version 13.1 (StataCorp LP, College Station, TX, USA) was used for statistical analysis.

## Results

One hundred fifty-nine patients who met the diagnostic criteria for APE were included. CTPA was the primary diagnostic tool (78 %), followed by ventilation and perfusion scintigraphy (8 %) and necropsy (5 %). Necropsy was reserved for unstable patients who died before performing an imaging test. The mean age of the patients was 58 ± 17 years; white race (79 %) and female gender (56 %) were predominant. The average PESI score was 100 ± 43 and its distribution among the risk stratification groups was: I – very low (20.7 %), II – low (20.7 %), III – moderate (19.5 %); IV – high (12.5 %); V – very high (26.4 %). Circulatory shock occurred in 16/159 (10 %) of patients, and cardiorespiratory arrest in 11/159 (7 %).

The average length of stay was 7 ± 17 days; during this period, anticoagulation with low molecular weight heparin was used in 67 % of patients, and anticoagulation with unfractionated heparin in 22 %. Thrombolysis was performed using alteplase in 37 patients (23 %). After hospital discharge, anticoagulation maintenance was performed with warfarin in 64 % and direct oral anticoagulants (rivaroxaban) in 9.5 %. The inferior vena cava filter was used in 2.5 % of cases. The in-hospital mortality rate was 15 %. Overall 30-day mortality was 20.7 % and 1-year mortality was 31 %. The other demographic, clinical, and laboratory characteristics of the included patients are shown in Table 3.

**Table 3:** Comparison of demographic, clinical, laboratorial and therapeutic features in hospitalized patients with acute pulmonary embolism divided according to the presence or absence of bleeding events and TIMI bleeding categories (major, minor and with attention required).

	Bleeding events			Bleeding category			
Features	Absent (*n* = 123)	Present (*n* = 36)	p	Major (*n* = 10)	Minor (*n* = 11)	Attention required (*n* = 15)	p
**Demographics**							
Male gender; n (%)	56 (45)	13 (36)	0.316	03 (30)	03 (27)	07 (46)	0.534
White people; n (%)	100 (81)	27 (75)	0.407	08 (80)	06 (54)	13 (86)	0.174
Age							
> 75-years, n (%)	22 (17)	05 (13)	0.574	01 (10)	01 (09)	03 (20)	0.801
> 65-years, n (%)	51 (41)	17 (47)	0.539	05 (50)	04 (36)	08 (53)	0.761
Age; years, mean ± SD	57.2 ± 17.3	58.6 ± 16.0	0.668	60.8 ± 14.7	56.8 ± 14.7	58.6 ± 18.4	0.926
**Clinical findings**							
Duration of symptoms; days, mean ± SD	6.8 ± 10.9	6.8 ± 9.3	0.995	5.7 ± 6.1	5.9 ± 9.0	8.2 ± 11.4	0.925
Dyspnea, n (%)	98 (79)	30 (83)	0.751	08 (80)	08 (72)	14 (93)	0.574
Hemoptysis, n (%)	10 (08)	05 (13)	0.306	02 (20)	01 (09)	02 (13)	‒
Syncope, n (%)	18 (14)	05 (13)	0.897	01 (10)	03 (27)	01 (06)	0.503
Cough, n (%)	38 (30)	12 (33)	0.804	04 (40)	03 (27)	05 (33)	0.929
Fever, n (%)	15 (12)	04 (11)	0.848	0 (0)	01 (09)	03 (20)	0.500
Pleuritic chest pain, n (%)	35 (28)	14 (38)	0.245	04 (40)	02 (18)	08 (53)	0.170
Any chest pain, n (%)	44 (35)	14 (38)	0.757	02 (20)	05 (45)	07 (46)	0.527
Cardiac arrest, n (%)	07 (05)	04 (11)	0.260	02 (20)	01 (09)	01 (06)	‒
Altered mental status, n (%)	27 (21)	10 (27)	0.467	04 (40)	03 (27)	03 (20)	0.599
Circulatory shock, n (%)	12 (09)	04 (11)	0.812	02 (20)	01 (09)	01 (06)	0.728
RR > 20 breaths/min, n (%)	81 (65)	25 (69)	0.644	05 (50)	06 (54)	14 (93)	0.190
HR > 100 bpm, n (%)	52 (42)	14 (38)	0.810	05 (50)	01 (09)	08 (53)	0.153
Signs of DVT, n (%)	35 (28)	12 (33)	0.592	02 (20)	05 (45)	05 (33)	0.591
PESI, categories			0.813				0.354
I, n (%)	26 (21)	07 (19)		0 (0)	02 (18)	05 (33)	
II, n (%)	24 (19)	09 (25)		04 (40)	04 (36)	01 (06)	
III, n (%)	26 (21)	05 (13)		01 (10)	01 (09)	03 (20)	
IV, n (%)	16 (13)	04 (11)		01 (10)	02 (18)	01 (06)	
V, n (%)	31 (25)	11 (30)		04 (40)	02 (18)	05 (33)	
PESI; mean ± SD	99.5 ± 43.9	103.4 ± 44.6	0.639	114.0 ± 40.6	96.8 ± 48.5	101.1 ± 46.0	0.784
RR; breaths/min, mean ±SD	24.3 ± 7.8	23.6 ± 6.5	0.597	23.4 ± 8.80	20.6 ± 5.9	25.4 ± 5.0	0.466
HR; bpm, mean ± SD	95.2 ± 20.1	95.4 ± 17.8	0.944	96.2 ± 12.6	85.6 ± 17.9	101.6 ± 18.6	0.260
SBP; mmHg, mean ± SD	122.0 ± 27.0	118.2 ± 30.4	0.489	118.3 ± 44.4	121.4 ± 29.0	115.9 ± 22.3	0.868
DBP; mmHg, mean ± SD	75.0 ± 15.9	74.9 ± 21.4	0.969	68.3 ± 28.8	78 ± 24.3	76.5 ± 13.3	0.616
SpO_2_; %, mean ± SD	90.3 ± 7.8	92.3 ± 5.3	0.239	90.0 ± 5.8	96.4 ± 1.14	92.0 ± 5.5	0.314
SI (HR/SBP), mean ± SD	0.82 ± 0.27	0.89 ± 0.40	0.211	0.97 ± 0.51	0.76 ± 0.30	0.94 ± 0.39	0.230
**Risk factors**							
Previous DVT, n (%)	24 (19)	10 (27)	0.287	01 (10)	04 (36)	05 (33)	0.287
Active malignancy, n (%)	10 (08)	03 (08)	0.969	01 (10)	01 (09)	01 (06)	‒
Recent surgery < 1-month, n (%)	20 (16)	04 (11)	0.438	0 (0)	02 (18)	02 (13)	0.315
Immobilization > 3-days, n (%)	31 (25)	11 (30)	0.522	04 (40)	04 (36)	03 (20)	0.589
Fracture, n (%)	16 (13)	04 (11)	0.763	01 (10)	01 (09)	02 (13)	0.973
Previous stroke, n (%)	16 (13)	04 (11)	0.763	01 (10)	01 (09)	02 (13)	0.973
Oral contraceptive use, n (%)	14 (11)	04 (11)	0.952	01 (10)	01 (09)	02 (13)	0.988
Obesity, n (%)	44 (35)	13 (36)	0.920	03 (30)	05 (45)	05 (33)	0.966
Heart failure, n (%)	16 (13)	04 (11)	0.763	0 (0)	02 (18)	02 (13)	0.619
COPD, n (%)	14 (11)	04 (11)	0.964	01 (10)	02 (18)	01 (06)	0.836
Thrombophilia, n (%)	04 (03)	08 (22)	0.001	01 (10)	04 (36)	03 (20)	‒
**Laboratory findings**							
Anemia, n (%)	53 (43)	18 (50)	0.487	06 (60)	06 (54)	06 (40)	0.663
Hemoglobin, g/dL, mean ± SD	12.7 ± 2.3	12.5 ± 2.0	0.661	12.3 ± 1.9	12.2 ± 2.2	12.8 ± 2.1	0.887
Platelets < 150 × 10^3^, n (%)	21 (17)	10 (27)	0.161	02 (20)	03 (27)	05 (33)	0.449
Platelets < 50 × 10^3^, n (%)	02 (1.6)	01 (2.0)	–	0 (0)	0 (0)	01 (06)	‒
Platelets ×10^3^ (/mm^3^), mean ± SD	223.684 ± 96.815	210.361 ± 98.743	0.471	250.300 ± 126.316	193. 909 ± 53.476	195.800 ± 102.451	0.416
Creatinine, mg/dL, mean ± SD	1.2 ± 0.5	1.2 ± 0.4	0.772	1.3 ± 0.4	1.1 ± 0.3	1.3 ± 0.5	0.837
Creatinine clearance*, mean ± SD	68.2 ± 28.1	61.6 ± 27.0	0.221	52.7 ± 22.3	67.2 ± 24.1	63.5 ± 31.6	0.388
Lactate, mg/dL, mean ± SD	2.4 ± 1.8	3.3 ± 4.1	0.176	2.3 ± 1.0	5.2 ± 6.6	2.3 ± 1.5	0.031
Arterial pH, mean ± SD	7.40 ± 0.1	7.37 ± 0.1	0.282	7.34 ± 0.1	7.34 ± 0.1	7.42 ± 0.07	0.231
PT (INR), mean ± SD	1.37 ± 0.88	1.58 ± 0.97	0.249	1.38 ± 0.55	1.96 ± 1.37	1.40 ± 0.75	0.239
Troponin, ng/mL, mean ± SD	0.16 ± 0.35	0.17 ± 0.38	0.900	0.16 ± 0.24	0.22 ± 0.54	0.12 ± 0.18	0.949
NT-pro-BNP, pg/mL, mean ± SD	3.546 ± 4.642	3.719 ± 3.630	0.878	4.162 ± 4.729	4.605 ± 3.979	2.327 ± 1.820	0.779
**Treatment**							
Thrombolysis, n (%)	26 (21)	11 (30)	0.240	03 (30)	04 (36)	04 (26)	0.633
UH, n (%)	25 (20)	11 (30)	0.197	04 (40)	03 (27)	04 (26)	0.499
LMWH, n (%)	81 (65)	27 (75)	0.329	05 (50)	08 (72)	14 (93)	0.102
Warfarin, n (%)	79 (64)	23 (63)	0.892	04 (40)	09 (81)	10 (66)	0.130
DOAC, n (%)	13 (10)	02 (1.6)	0.354	01 (10)	0 (0)	01 (06)	‒
**Echocardiographic findings**							
RV dilatation	55 (44)	21(58)	0.102	07 (70)	07 (63)	07 (46)	0.402
RV failure	30 (24)	06 (16)	0.243	02 (20)	02 (18)	02 (13)	0.714
Acute pulmonary hypertension	57 (46)	21 (58)	0.177	07 (70)	06 (54)	08 (53)	0.509
Estimated pulmonary artery pressure, mmHg, mean ± SD	51.3 ± 24.6	54.2 ± 14.3	0.598	59.5 ± 5.2	48.0 ± 22.5	55.2 ± 6.8	0.717

SD, Standard Deviation; RR, Respiratory Rate; HR, Heart Rate; DVT, Deep Vein Thrombosis; PESI, Pulmonary Embolism Severity Index; SBP, Systolic Blood Pressure; DBP, Diastolic Blood Pressure; SpO_2_, Peripheral Oxygen Saturation; SI, Shock Index; COPD, Chronic Obstructive Pulmonary Disease; PT, Prothrombin Time; UH, Unfractionated Heparin; LMWH, Low Molecular Weight Heparin; DOAC, Direct Oral Anticoagulants; RV, Right Ventricle.

The prevalence of any bleeding was 36/159 (23 %), of major bleeding was 10/159 (06 %), of minor bleeding was 11/159 (06 %), and of bleeding requiring attention was 15/159 (10 %) during hospitalization.

Regarding clinical characteristics, a higher prevalence of thrombophilia was observed in patients with some bleeding than those without bleeding (22 % vs. 03 %; *p* = 0.001). Higher lactate levels were also observed in patients with minor bleeding compared to patients without bleeding (5.2 ± 6.6 mg/dL vs. 2.4 ± 1.8 mg/dL; *p* = 0.031). No other statistically significant differences were observed between demographic, clinical, laboratory, and treatment-related characteristics between patients who experienced bleeding and those without bleeding (Table 3).

The occurrence of any bleeding, bleeding requiring attention, minor bleeding, and major bleeding was not associated with 30-day overall mortality (*p* > 0.05 for all comparisons). However, major bleeding was associated with higher mortality in one year of follow-up with a Relative Risk (RR) of 2.00 (95 % CI 1.16–3.57; *p* = 0.044) (Table 4).

**Table 4:** Association with different types of bleeding according to TIMI (Thrombolysis in Myocardial Infarction bleeding risk) classification and 30-day and 1-year overall mortality.

Bleeding category (TIMI)	Outcome		
	30-day mortality		
	RR	95 %CI	p
Any bleeding	1.28	0.65 – 2.50	0.475
Required attention	1.49	0.66 – 3.34	0.351
Minor	0.42	0.06 – 2.79	0.322
Major	2.05	0.90 – 4.70	0.121
	**One-year mortality**		
Any bleeding	1.46	0.91 – 2.36	0.133
Required attention	1.59	0.90 – 2.80	0.142
Minor	0.56	0.15 – 2.00	0.326
Major	2.00	1.16 – 3.57	0.044

RR, Relative Risk; 95 % CI, 95 % Confidence Interval.

Table 5 shows the classification of patients according to the bleeding predictive scores (RIETE, Kuijer; PE-SARD; VTE-BLEED and ATRIA). Only the VTE-BLEED score showed statistically different discrimination between the groups, with 56/123 (46 %) patients being classified as high risk in the non-bleeding group versus 23/36 (64 %) who were classified as high risk in the group with bleeding (*p* = 0.05).

**Table 5:** Bleeding risk categories according to different bleeding prediction scores in patients with acute pulmonary embolism.

	Bleeding events			Bleeding category			
Bleeding risk score	Absent (*n* = 123)	Present (*n* = 36)	p^a^	Major (*n* = 10)	Minor (*n* = 11)	Attention required (*n* = 15)	p^b^
RIETE							
Low and intermediate, n (%)	113 (92)	33 (92)	0.969	09 (90)	10 (90)	14 (93)	0.847
High, n (%)	10 (08)	03 (08)		01 (10)	01 (09)	01 (07)	
Mean ± SD	2.41 ± 1.34	2.70 ± 1.31	0.254	3.05 ± 1.18	2.68 ± 1.50	2.50 ± 1.28	0.510
Kuijer							
Low, n (%)	26 (21)	29 (80)	0.826	01 (10)	02 (18)	04 (27)	0.838
Intermediate and high, n (%)	97 (79)	07 (20)		09 (90)	09 (82)	11 (73)	
Mean ± SD	1.62 ± 1.16	1.86 ± 1.25	0.290	1.96 ± 0.97	2.01 ± 1.29	1.69 ± 1.44	0.643
PE-SARD							
Low, n (%)	41 (33)	10 (28)	0.297	3 (30)	3 (27)	4 (26)	0.355
Intermediate, n (%)	62 (51)	16 (44)		3 (30)	4 (36)	9 (60)	
High, n (%)	20 (16)	10 (28)		4 (40)	4 (36)	2 (13)	
Mean ± SD	1.52 ± 1.37	1.75 ± 1.52	0.409	1.85 ± 1.84	2.13 ± 1.53	1.40 ± 1.29	0.482
VTE-BLEED							
Low, n (%)	67 (54)	13 (36)	0.05	2 (20)	4 (36)	7 (47)	0.141
High, n (%)	56 (46)	23 (64)		8 (80)	7 (64)	8 (53)	
Mean ± SD	2.21 ± 1.73	2.66 ± 1.52	0.161	3.2 ± 1.65	2.54 ± 1.66	2.36 ± 1.51	0.300
ATRIA							
Low, n (%)	98 (80)	27 (75)	0.553	8 (80)	8 (73)	11 (73)	0.903
Intermediate and high, n (%)	25 (20)	09 (25)		2 (20)	3 (27)	4 (27)	
Mean ± SD	2.08 ± 2.12	2.38 ± 1.79	0.432	2.60 ± 1.71	2.18 ± 1.83	2.4 ± 1.92	0.843

a Comparison between absent vs. present bleeding.

b Comparison among absent vs. major vs. minor vs. require attention bleeding events.

**Fig. 1:**
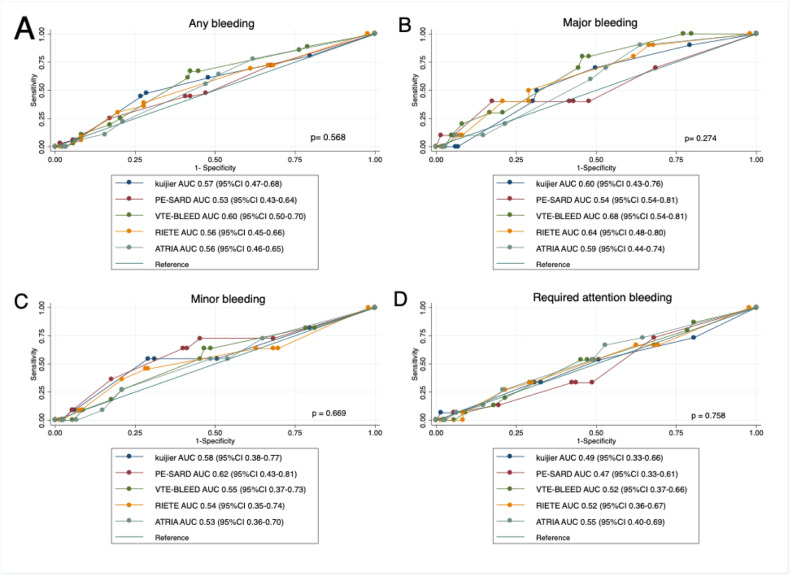
Receiver Operating Characteristic Curve (ROC) depicting the accuracy of different scores in predicting any bleeding (A), major bleeding (B), minor bleeding (C) and bleeding that required attention (D) in patients hospitalized with acute pulmonary embolism in Brazil. AUC, Area Under the Curve; 95 % CI, Confidence Interval of 95 %.

The VTE-BLEED score showed the highest accuracy for determining any bleeding with an AUC-ROC of 0.60 (95 % CI 0.50‒0.70) and for major bleeding with an AUC-ROC of 0.68 (95 % CI 0.54‒0.81); however, with no statistically significant difference compared to the other scores. All scores assessed showed an unsatisfactory performance for determining bleeding in this sample, as shown in Fig. 1.

## Discussion

This investigation showed a high prevalence of bleeding during hospitalization for APE in Brazil. Furthermore, major bleeding was associated with higher one-year mortality. Bleeding predictive scores showed unsatisfactory performance in identifying patients at higher risk of bleeding.

Kresoja et al. showed an in-hospital incidence of major bleeding of 3.5 % in patients with APE. Furthermore, patients with major bleeding had a higher risk of death within one-year follow-up with a Relative Risk (RR) of 3.6 (95 % CI 2.0–6.6; *p* < 0.001).^16^ Budaj-Fidecka et al. reported an incidence of major bleeding of 2.4 % and any bleeding of 6 % in individuals with APE during a three-month follow-up. In this same time, there was higher mortality in individuals who had major bleeding with a RR of 2.75 (95 % CI 1.29–5.87; *p* = 0.009).^17^ Compared to these two studies, the present investigation showed a higher in-hospital bleeding rate (6 % of major bleeding and 23 % of any bleeding). However, similar to these two reported studies, major bleeding was associated with a higher one-year mortality with an RR of 2.00 (95 % CI 1.16–3.57; *p* = 0.044).

An important detail that must be highlighted is the lack of standardization in the definition and classification of bleeding between studies. Several classifications are used for this purpose. This study used the TIMI classification for bleeding^15^; however, currently, the International Society on Thrombosis and Haemostasis (ISTH) criteria is the most used.^18^ In this last classification, major bleeding is considered to be any fatal bleeding and/or symptomatic bleeding in a critical area or organ, such as intracranial, intraspinal, intraocular, retroperitoneal, intra-articular or pericardial, or intramuscular with compartment syndrome, and/or bleeding causing a fall in hemoglobin levels > 2.0 g/dL or leading to a transfusion of 2 units or more of whole blood or red cells. If the authors had used this last classification, the prevalence of major bleeding would have been even higher in the present study.

The higher prevalence of bleeding in this study could be explained in part by the greater severity of patients hospitalized with APE in Brazil. In a cohort of 1880 individuals with APE, Pollack et al. showed an overall 30-day mortality of 5.4 %.^19^ Laporte et al. showed a 30-day mortality of 3.3 % in a sample of 6518 patients diagnosed with APE.^20^ In the present investigation, the authors observed an in-hospital mortality of 15 %, a 30-day mortality of 20.7 %, and a one-year mortality of 31 %. The incidence of circulatory shock upon hospital admission was higher in the present study's sample (10 %) when compared to studies by Pollack et al. (3.0 %; *p* = 0.002) and Laporte et al. (3.80 %; *p* = 0.006).^19^^,^^20^ Furthermore, our study included individuals with higher PESI scores (100 ± 43 vs. 88 ± 34; *p* = 0.0001) compared with Pollack's sample.^19^

In a cohort of 727 Brazilian patients with APE, Volschan et al. showed an in-hospital mortality of 19.5 % and circulatory shock prevalence of 19.9 %.^21^ In another Brazilian study, Soriano et al. also observed high circulatory shock and 30-day mortality rates, respectively 11 % and 23 %. In this last study, 59 % of the sample presented PESI > 84-points.^22^ Probably, only the most severe APEs are diagnosed and hospitalized in Brazil, which ends up contributing to the higher bleeding in these patients.

Regarding predictive bleeding scores, Klok et al. evaluated the Kuijer, RIETE, HEMORR2HAGES, HAS-BLED, and ATRIA scores to predict bleeding within 30-days in individuals with APE. As in the present investigation, the prognostic performance for predicting bleeding was limited with an AUC-ROC of 0.57 to 0.64.^7^ In agreement, Zhu Zhang et al. showed an unsatisfactory accuracy of the Kuijer and RIETE scores for predicting major bleeding within three months after APE diagnosis with an AUC-ROC of 0.57 and 0.56; respectively.^8^

In a study to externally validate the PE-SARD score, 50,686 individuals with APE were included, Chopard et al. showed an AUC-ROC of 0.65 for this score in discriminating patients at risk of major bleeding within 30-days.^6^ Kresoja et al. reported an AUC-ROC of 0.69 for the VTE-BLEED score in predicting major bleeding in patients hospitalized with APE.^16^

Some limitations deserve to be highlighted. First, the study was unicentric and carried out in a highly complex tertiary referral hospital. This may have affected the findings by introducing a selection bias, leading to the inclusion of patients with greater severity and comorbidities. Second, the bleeding classification was carried out exclusively using the TIMI. Because of this, the comparison with other studies is difficult and it may have even underestimated the major bleeding in the present investigation. Third, this study was carried out when warfarin was the primary anticoagulant used in the long term; only 9.5 % of patients used Direct Oral Anticoagulants (DOACs), which may have contributed to the higher bleeding risk.

To our knowledge, this is the only study that evaluated the prevalence of bleeding and the impact of this complication on mortality during APE treatment in the Brazilian population. Furthermore, no validation study of predictive bleeding scores in patients with APE in Brazil was found.

In conclusion, the authors observed a high prevalence of bleeding in this sample of Brazilian patients hospitalized with APE. The presence of major bleeding was associated with a higher mortality within one year. The evaluated bleeding predictive scores showed unsatisfactory performance in identifying patients at high risk of bleeding. Therefore, the authors do not recommend using these scores when making decisions regarding antithrombotic therapy for these patients. National, multicenter, and prospective registries are necessary to more accurately determine the prevalence of bleeding in hospitalized patients with APE in Brazil and to develop appropriate bleeding predictive scores for this population.

## Conflicts of interest

The authors declare no conflicts of interest.
